# An Examination of the Role of Mentees’ Social Skills and Relationship Quality in a School‐Based Mentoring Program

**DOI:** 10.1002/ajcp.12397

**Published:** 2019-09-25

**Authors:** Loïs Schenk, Miranda Sentse, Margriet Lenkens, Gera E. Nagelhout, Godfried Engbersen, Sabine Severiens

**Affiliations:** ^1^ Department of Psychology, Education and Child Studies Erasmus University Rotterdam Rotterdam The Netherlands; ^2^ Institute of Criminal Law and Criminology Leiden The Netherlands; ^3^ Erasmus Medical Centre Rotterdam The Netherlands; ^4^ IVO Research Institute The Hague The Netherlands; ^5^ Department of Health Promotion and Department of Family Medicine Maastricht University (CAPHRI) Maastricht The Netherlands; ^6^ Department of Public Administration and Sociology Erasmus University Rotterdam Rotterdam The Netherlands

**Keywords:** Social skills, Youth mentoring, Relationship quality, School‐based mentoring

## Abstract

Research on youth mentoring highlights the importance of the relationship quality between mentor and mentee; mentoring results in more positive outcomes when the mentee perceives the relationship as satisfying and trustworthy. Research on relationship quality shows that social skills are important for constructing new relationships. However, whereas improved social skills are often one of the main goals of youth mentoring, little is known about the importance of social skills for relationship quality in youth mentoring relations. In this study, we examined whether mentee's pre‐intervention social skills were related to mentor–mentee relationship quality as perceived by the mentee, and in turn, if relationship quality was associated with post‐intervention social skills. We additionally examined possible gender and age differences in these associations. Data were used from a two‐wave study that assessed relationship quality and social skills before and after one semester of mentoring of 390 secondary school students in a school‐based mentoring program. Results indicated that relationship quality was positively associated with post‐intervention social skills. However, only for young mentees pre‐intervention social skills were associated with better relationship quality. Moreover, only for young mentees, relationship quality mediated the association between pre‐ and post‐intervention social skills.

## Highlights

- Mentees’ perceived relationship quality is positively associated to social skill outcomes.
- Young mentees’ pre‐social skills are positively associated to mentor‐mentee relationship quality.
- Relationship quality only mediates young mentees’ pre and post‐social skills.
- Relationship quality is equally important for boys and girls in changes in social skills.

## Introduction

Supportive relations during adolescence are important for youths in their transition to adulthood. Many adolescents have a network in which supportive adults and peers are present, but for some adolescents the existing network is not strong or diverse enough to help navigate through the social and academic challenges in their lives (Raposa, Erickson, Hagler & Rhodes, 2018). In these cases, mentors can serve as additional sources of support and guidance. Indeed, mentoring‐based interventions are widely used and have become increasingly popular in improving academic, behavioral, and health domain outcomes. Via mentoring, youths’ social networks are expanded by the commitment of someone other than their parents, who is willing to meet on a structural basis, and who ensures that the mentee's well‐being is central to the relationship.

Whereas most relationships with adults arise through organic social connections, such as with family friends and neighbors, many adolescents have reduced access to these connections and may benefit from formal mentoring programs (Hagler & Rhodes, 2018; Raposa et al., 2018). Schools provide a primary context to foster mentoring relationships outside of the youth's family. School‐based mentoring is one of the fastest growing forms of mentoring, in which volunteers meet their mentee regularly in a school‐setting. It is a low‐cost way to support disadvantaged students by providing a positive tie, and this relationship may be helpful when students experience social and academic difficulties (Herrera & Karcher, 2013). A major advantage of school‐based mentoring is that it reaches youth who otherwise are less likely to take part in a mentoring program, for instance because their parents are not able or willing to take initiative to sign up their child for a community‐based mentoring program (Herrera & Karcher, 2013).

In Rhodes’ (2005) proposed model of youth mentoring, positive outcomes of mentoring take place in three developmental areas: socio‐emotional, cognitive, and identity‐related. To illustrate, as part of social and emotional development, mentees’ social skills may increase through mentoring. Positive experiences in a mentor relationship, and mentors providing a model of effective communication, may enable youth to interact more effectively with parents and peers. In this way, mentoring furthers youths’ socio‐emotional development including their social skills. However, beneficial effects in these areas are only expected when there is a strong relationship characterized by mutuality, trust, and empathy between mentor and mentee. In this model, both a strong relationship and the pathway of this relation to positive outcomes are conditioned by mentees’ individual and contextual influences. Mentees’ interpersonal history and social competencies, for example, are theorized to affect relationship quality, but also affect the way relationship quality leads to positive youth outcomes.

Although mentoring is becoming a widespread intervention, it still leaves room for improvement. Meta‐analyses of mentoring programs revealed only small to modest effects of mentoring on emotional, behavioral, and educational domains (DuBois, Portillo, Rhodes, Silverthorn, & Valentine, 2011; Eby et al., 2013). However, in both community‐ and school‐based mentoring, an important facilitating condition to increase effect sizes is mentor–mentee relationship quality (DuBois et al., 2011; Herrera, Grossman, Kauh, Feldman, & McMaken, 2007). Further research is necessary to gain more insight into how and for whom relationship quality is important in mentoring interventions.

Mentoring‐related improvements may have far‐reaching effects. Developing social skills, for example, is one of the main goals of mentoring (DuBois, Holloway, Valentine, & Cooper, 2002) and can result in more competence to construct new supportive relationships with peers, parents, and other adults. Yet, as proposed in Rhodes’ (2005) conceptual model, in order to build and benefit from a supportive mentor relationship, one can reason that some minimal level of social skills is required from the mentee. For example, being able to formulate one's needs is necessary to receive the right support, also known as “proto‐professionalism” in health care (De Swaan, 1990). Not surprisingly, it has been shown that mentor–mentee relationship quality is one of the main factors that facilitate positive outcomes of mentoring (Bayer, Grossman, & DuBois, 2013; Eby et al., 2013; Rhodes, 2005), such as improved social skills. However, it is unclear to what extent mentee's social skills before mentoring are related to mentor–mentee relationship quality and how the latter is related to social skills after mentoring. If it is true that relatively high levels of social skills prior to mentoring are necessary for youths to benefit from mentoring, then the mentees with relatively low levels of social skills who might need mentoring the most, needs consideration. As such, in this study we investigate the possible mediating role of mentor–mentee relationship quality between mentees’ social skills before and after mentoring, elucidating how and for whom mentoring can be potentially more successful.

### Quality of Mentoring Relationships

It seems unlikely that positive dynamics unfold in a mentoring relationship without the feeling of connection. For mentees to learn, imitate, and share feelings with their mentor, relationship qualities such as trust, empathy, sensitivity, and attunement should be present (Rhodes, Spencer, Keller, Liang, & Noam, 2006). When a close and trusting relationship does not develop, youth and mentors may both disengage from the match before the appearance of positive outcomes. Even when the relationship does continue, it hinders the way mentees can open up, share, and learn from their mentor. Other definitions of relationship quality in the mentoring literature include mentees’ feelings toward the mentor, satisfaction with the relationship, and liking (Eby et al., 2013), perceived mutuality (Rhodes et al., 2006), and affinity and closeness (DuBois et al., 2011). Eby et al.'s (2013) meta‐analysis showed that higher relationship quality in mentoring increased psychosocial and instrumental support, and that it was one of the most important predictors of successful outcomes.

In particular, research on school‐based mentoring shows similar results. The benefits of school‐based mentoring were assessed in a randomized‐controlled trial of over 1,000 students (Bayer et al., 2013). Evidence was found for a close mentoring relationship being the key to effectiveness in school‐based mentoring. Surprisingly, school‐based mentoring programs that focused solely on academic outcomes, had similar effects on academic outcomes as relationship‐only programs, illustrating the major role of relationship quality for a broad range of mentoring outcomes. Moreover, a recent evaluation of the effects of school‐based mentoring in the United States showed not only that higher mentor–mentee relationship quality led to desired outcomes, but also that when relationship quality was low the opposite was true, that is, it was associated with harmful effects such as misconduct (Lyons & McQuillin, 2019). Studying possible determinants of relationship quality in mentoring thus seems to be of considerable relevance for improving mentoring research and practice, because it is one of the critical components of effective mentoring.

### Social Skills and Interpersonal Relationships

In this study, social skills are studied as one of the possible determinants and outcomes of relationship quality. Socials skills pertain to interacting with others in an appropriate and effective way (Segrin, 1992). Individuals with social skills attract social attention, are more liked due to interpersonal attraction, provoke more positive responses, and are more active and effective in social interactions (Segrin & Taylor, 2007). As a result, social skills are strongly related to the establishment and maintenance of positive and supportive relations with others (Segrin & Taylor, 2007).

Social skills are also an important factor in decreasing risk behaviors. For youths being at risk due to economic disadvantage or emotional and behavioral problems, social skills are an individual characteristic found critical in counteracting negative effects of risk exposure (Domitrovich, Durlak, Staley, & Weissberg, 2017). Moreover, social skills become more important during adolescence. Whereas in childhood and pre‐adolescence parents fulfill children's social needs, the focus in adolescence redirects to friends. This demands more interpersonal competencies in more mature forms of close relationships. Research showed that social competence and relationship quality in friendship among adolescents are consistently related and appear to be of great importance for adolescents (Buhrmester, 1990; Cillessen, Jiang, West, & Laszkowski, 2005).

Theories that explain the relation between socials skills and the quality of relationships merely focus on these factors in relation to psychological distress. Irrespective of this context, the theories offer useful insights in how social skills and relationship quality are related. The *social skills deficit vulnerability model,* for example, theorizes that individuals with poor social skills are more vulnerable to the development of psychological distress because they have less protective social support (Segrin, McNelis, & Swiatkowski, 2016). The lack of effective mechanisms for coping with stress may contribute to the development of psychological distress, whereas individuals with well‐developed social skills experience more protection during difficulties. This relation between social skills and psychosocial problems is assumed to be mediated by the access and deployment of social support (Segrin et al., 2016).

Social skills allow for positive interpersonal relationships, and in line with the above, the possession of social skills may also be beneficial in constituting relationships for youths in mentoring relations. Research suggests that relationship quality in mentoring depends on mentees’ ability to form a close relationship (e.g., DuBois et al., 2011; Eby et al., 2013; Rhodes et al., 2006). Although in several studies it was found that mentees with more relational experience report higher relationship quality (Bayer et al., 2013; Eby et al., 2013), other research fails to find an association between relational experience and perceptions of mentees’ relationships with their mentors (Schwartz, Rhodes, Chan, & Herrera, 2011). From these studies, it also remains unclear whether relational experience is related to youth's social skills or to their limited accesses to supportive others, or both.

Besides the association between social skills and relationship quality, relationship quality can be, subsequently, associated with mentoring outcomes. Mentees with social skills are expected to be able to derive more benefits from their mentor relationship than less socially skilled youth (DuBois et al., 2011). To illustrate, a study on a school‐based mentoring program showed that youths with moderately strong relationships at baseline had greater improvements in overall academic performance and classroom effort from mentoring, compared to relationally vulnerable mentees (Schwartz et al., 2011). Expecting mentees’ higher baseline social skills to be related to better outcomes of social skills through higher relationship quality, raises an important issue in mentoring. The phenomenon, that individuals with the richest resources are to benefit most from new experiences and also at a faster rate, is referred to as the Matthew Effect (Merton, 1988). Youths, with previous experiences of close relationships with a non‐familial adult, are likely to develop more social skills compared to youths who lack this experience. These socially skilled youths, then, are able to leverage their social skills to establish a high‐quality relationship in mentoring. Consequently, through this high relationship quality, these youths will profit the most from mentoring, that is, their social skills increase more and faster compared to less socially skilled youths. This cumulative advantage eventually may lead to a wider gap between students with poor and excellent social skills (DiPrete & Eirich, 2006). As such, social skills are hypothesized to positively influence the mentor–mentee relationship quality, which in turn should lead to a further improvement in social skills. To date, however, empirical evidence of the proposed relation between socials skills of mentees and the relationship quality with their mentor seems absent in the mentoring literature.

### Age and Gender

Research has extensively focused on age and gender differences in mentoring outcomes; however, little research has focused on the age and gender differences in the process of mentoring (Liang, Bogat, & Duffy, 2013). Based on developmental and social psychology literature, we explore possible differences in the proposed relations between social skills and relationship quality according to the age and gender of the mentees.

Social skills increase as a consequence of neurological maturation during adolescence (Crone & Dahl, 2012). Older mentees will therefore have higher levels of social skills. Subsequently, due to faster neurological maturation for girls and western socialization patterns, gender differences in social skills increase in adolescence (Silberman & Snarey, 1993). In addition to gender differences in adolescents’ social skills, appreciation of mentoring relationships differs among boys and girls as well. These differences may influence the way boys and girls perceive the quality of their mentoring relationship. To illustrate, in a sample of 1,138 youths in a Big Brother Big Sister mentoring program, girls in short (1–6 months) and medium (7–12 months) lasting mentor relationships were less satisfied with the relationship than boys. In long‐term relationships, however, girls were more satisfied than boys (Rhodes, Lowe, Litchfield, & Walsh‐Samp, 2008). Given that our sample is drawn from a short‐term mentoring program, we expect girls to be less satisfied with the relationship quality compared to boys.

As for the associations between social skills and relationship quality, there may be differences as well. For boys, engaging in activities is a way to establish relationship quality, whereas for girls, self‐disclosure is considered as a sign of relationship quality (Pollack, 1999). Social skills, therefore, seem to be a more likely precondition to establish relationship quality for girls than for boys. Additionally, research suggests that for girls, the quality of a relationship is more likely to be related to outcomes than for boys. Girls may both benefit from or be harmed by the relationship quality in mentoring. To illustrate, Karcher (2008) found girls in school‐based mentoring to benefit from mentoring, only when there was high relationship quality. In our study, relationship quality may be a stronger predictor of social skills outcomes for girls than for boys. Based on this, we explored possible gender and age differences in the associations between social skills and relationship quality.

### The Present Study

Social skills research showed that more social skills allow for more satisfying and trusting relationships. How this is the case in mentoring relationships, remains unclear, while insight in the role of individual characteristics in establishing high‐quality mentoring relationship is necessary to improve mentoring outcomes. In doing so, existing mentoring programs can take the mentees’ social skills into account while matching, or pay more attention to the skills needed to develop positive relations prior to the start of a mentoring relation. The overall aim of the present study is to see how social skills before mentoring, relationship quality, and social skills after mentoring are related in a school‐based, short‐term mentoring program. We will formally test the mediating role of relationship quality between pre‐social skills and post‐social skills and hypothesize that (a) mentees’ pre‐social skills are positively related to relationship quality of mentoring relationships and that (b) better relationship quality, in turn, is related to more post‐social skills. We will additionally examine whether these associations differ between ages and gender.

## Method

### Participants

Participants were drawn from the Mentors of Rotterdam program, which is the largest school‐based mentoring program of the Netherlands. The program provides mentors from The Rotterdam University of Applied Sciences to classes at seven high schools in Rotterdam South. This area has the highest ethnic and cultural diversity, the lowest socioeconomic status score, and the largest concentration of young people in the city of Rotterdam (Van den Berg, Schouten, Smit, & Van Veelen, 2014). In the Netherlands, and in large cities such as Rotterdam in particular, migration has changed the ethnic landscape, resulting in so‐called minority–majority cities, also described as “superdiversity” (Vertovec, 2007). For example, in many neighborhoods, more than 50% of the youth population is of second‐generation immigrant background (Crul, Schneider, & Lelie, 2013). This group of migration youth itself is diverse in terms of educational levels, socioeconomic backgrounds, religion, et cetera. Ethnicity, in this sense, is no longer relevant in describing the population. The mentoring program adopts this idea of superdiversity and, therefore, does not approach diversity in terms of ethnic and cultural differences only. Aside from the diversification of diversity, growing up in a superdiverse context, such as Rotterdam South, is often still a risk factor for youths who are vulnerable for, among others, school dropout and school absenteeism (Vertovec, 2007). The composition of this area is reflected in the pupil population of the schools. In addition, in Rotterdam South a high level of school segregation is present (Sykes & Kuyper, 2013). This means that children in Rotterdam South whose parents are higher educated than the rest of the population, and/or are native Dutch, go to schools in different parts of the city. This leads to an uneven distribution of students along ethnic and social lines at schools in Rotterdam South. Attending schools with lower levels of socioeconomic status on average is related to lower academic achievement (Sykes & Kuyper, 2013). Accordingly, youths from high schools in Rotterdam South have lower school results compared to the national level, and higher percentages of school dropout and youth unemployment (De Boom, Roode, van Wensveen, & de Graaf, 2017). We consider these characteristics of the area as an indication of the relative disadvantaged population of the schools in our study. Schools in the Netherlands are no longer allowed to register ethnic background of their students. Therefore, ethnicity and socioeconomic status, at school and individual level in our sample, were not available.

Mentors were second‐year students from the Rotterdam University of Applied Sciences (most students are 18 or 19 years old at this time), who could serve as a mentor in the program as an optional course during their course program. They came from a broad range of programs (e.g., social sciences and math). Given the diverse student composition of this university, we assume that the mentor population was ethnically/culturally diverse, and that the age difference between mentor and mentees was no more than 6 years (Rotterdam University of Applied Sciences, 2015). One‐on‐one matching was done based on common interests and attitudes; factors proven to be essential for an effective match (Sipe, 2002). Mentors and mentees filled out a form relating to personal characteristics, hobbies, and qualities. Teachers then matched mentors and mentees based on these forms. Mentoring activities were fun‐focused (playing games, cooking, sports), academic‐focused (planning and homework, career guidance), and interpersonal‐focused (talking about personal lives). The aim of these activities was to improve grades, offer career guidance, and support social–emotional development, through a trusting bond, role modeling, successful experiences, and study skills. Mentoring took place one‐on‐one, once a week for an hour, at school. In a few cases, not enough mentors were available, so that mentoring took place one on two. Mentors received training prior to the mentoring program and had weekly intervision and supervision meetings.

### Procedure

In the first school year (2015‐2016), a total of 240 students from 16 classes were assigned a mentor and received mentoring for at least one semester. In the second year of the program (2016‐2017), 356 students from 21 classes were assigned a mentor. All students of the selected classes received mentoring, and this was a total of 596 students (mentees). Participation in the study was completely voluntary. Mentees filled out a *student survey* at baseline (the start of the semester) and at follow‐up (6 months later). Questions in this student survey addressed mentees’ self‐efficacy, school belonging, social skills, and career orientation. Additionally, mentees filled out a survey about their mentoring relationship afterward (*mentee survey*). In this survey, relationship quality was assessed.

The present study focused on social skills as measured in the student survey, and its association with relationship quality as measured in the mentee survey. Data from both years were merged in one dataset (*n* = 596). A few students, however, received mentoring in two semesters and were duplicate cases (*n* = 28). We only included the data of the first mentoring semester of these students. Students were included when they filled in the survey at baseline and at follow‐up. A total of 390 mentees fulfilled these conditions (45.65 percent boys; *M*_age_ = 13.19 years, *SD *=* *1.47). Mentees who did not meet these criteria were compared to the final sample, in order to assess possible sample bias. *t* Tests revealed that these groups did not differ significantly in pre‐social skills and relationship quality, *t*(387) *=* 0.78, *p *=* *.44 and *t*(466) = 0.78, *p *=* *.44, respectively. However, students who completed all the surveys had significant higher post‐social skills than students who did not, *t*(457) = −2.34, *p = *.02.

### Measures

#### Social Skills

Social skills of mentees were assessed by twelve statements that mentees were asked to rate according to the level of agreement. Scores ranged from 1 (*not true at al*l) to 5 (*totally agree*). Six out of the twelve items were formulated negatively; thus, these items were recoded. The items of these scales were very similar to the Matson Evaluation of Social Skills with Youngsters containing various aspects of social skills (Matson, Rotatori, & Helsel, 1983) and were previously used in research on mentoring in the Netherlands (Klooker & Boswinkel, 2013). Aspects of social skills were peer acceptance (e.g., “Most classmates like me”), dealing with conflict (e.g., “I often argue”), and prosocial behavior (e.g., “I have a chat easily”). We took the mean of these twelve items to create scale scores for social skills.

Social skills were measured at baseline and at follow‐up. This resulted in two variables we called “pre‐social skills” and “post‐social skills.” The first variable refers to social skills before the mentoring, the second to social skills after mentoring. Cronbach's α of social skills variables at the two measurements were .75 and .79. Higher scores on these variables reflect higher levels of social skills.

#### Mentoring Relationship Quality

The relationship quality scale was developed for the present study and contained six items corresponding to trust (e.g., “I trust my mentor”) and role modeling (e.g., “I consider my mentor as an example”) in the mentoring relationship. Mentees completed the relationship quality measure. Scores ranged from 1 (*not true at all*) to 5 (*totally agree*). We used the mean of the scores to construct the relationship quality variable. Cronbach's α of this scale was .90, and higher scores on this scale reflect higher relationship quality.

### Analysis

We used *t* tests to examine possible age and gender differences in the mean levels of our study variables. Bivariate associations between the variables were explored with Pearson correlations. Then, we estimated a series of mediation analyses to test for the direct and indirect paths between social skills and mentor–mentee relationship quality.

First, we tested a mediation model for the overall sample, using the PROCESS macro in SPSS (Hayes, 2015). We tested whether relationship quality mediated the association between social skills pre and post mentoring. This model was run with age and gender as covariates. Second, we examined differences between age and gender in this model. More specifically, we tested “the when of the how” (Hayes, 2015). This means we looked at the mediating role of relationship quality (how), separately for boys and girls, and separately for younger and older mentees (when). As such, two additional mediation analyses were run with age and gender as moderator, respectively. To determine the age and gender differences in the conditional indirect effects, we generated bootstrap confidence intervals. To interpret interaction effects, we used simple effect tests in PROCESS for each group of the moderator.

## Results

### Descriptive Statistics

Table 1 contains the means and standard deviations of the study variables for the overall sample and for boys and girls separately. *t* Tests showed that boys and girls did not significantly differ in pre‐social skills (*t*(370) = 0.08, *p *=* *.94), but girls reported slightly more social skills post mentoring than boys *t*(377) = −2.13, *p *=* *.03). There were no significant gender differences in mentor–mentee relationship quality (*t*(377) = −1.53, *p *=* *1.28). When mentees were compared on age with a cutoff on the mean age (not in tables), younger (than mean age) mentees reported significantly more pre‐social skills as compared to older mentees (*M *=* *4.09, *SD *=* *0.48, *M *=* *3.97, *SD *=* *0.47, respectively), *t*(370) = −2.46, *p *=* *.01. The same differences were found in post‐social skills, with social skills of younger mentees (*M *=* *4.02, *SD *=* *0.45), being significantly higher than those of older mentees (*M *=* *3.92, *SD *=* *0.46) *t*(377) = −2.13, *p *=* *.03. No significant differences were found in relationship quality for younger and older mentees (*M *=* *3.88, *SD *=* *0.78, *M *=* *3.79, *SD *=* *0.87, respectively), *t*(377) = −1.10, *p *=* *.27.

**Table 1:** Means, standard deviations, and *t* tests

Variable	All (*n* = 390)		Girls (*n* = 206)		Boys (*n* = 173)			Gender differences	
	*M*	*SD*	*M*	*SD*	*M*	*SD*	Min‐Max	*t‐*value	*p*
Age	13.19	1.47	13.16	1.49	13.23	1.45	11–19	0.46	.65
Relationship quality	3.86	0.82	3.91	.811	3.78	.83	1–5	−1.53	.13
Pre‐social skills	4.05	0.48	4.04	.49	4.04	.46	1–5	0.08	.94
Post‐social skills	3.98	0.46	4.02	.44	3.92	.47	1–5	−2.13	.03

Results of the Pearson correlations in the overall sample indicated a significant positive association between mentor–mentee relationship quality and both pre‐ and post‐social skills, (*r = *0.11, *p *<* *.05, and *r *=* *0.19, *p *<* *.01, respectively). For girls, pre‐ nor post‐social skills were correlated with relationship quality. For boys, relationship quality was significantly positively correlated with pre‐social skills (*r = *0.17, *p *<* *.05) and with post‐social skills (*r *=* *0.28, *p *<* *.01). For older mentees (age > 13.19), there were no significant correlations between the study variables. For younger mentees (age < 13.19), however, relationship quality correlated with pre‐social skills (*r *=* *0.21, *p *<* *.01), and relationship quality correlated with post‐social skills (*r *=* *0.28, *p *<* *.01).

### Mediation Analyses

#### Overall Sample

First, we tested for the overall sample whether the relation between social skills pre and post mentoring was mediated by mentor–mentee relationship quality, while controlling for gender and age. The results of this mediation analyses are reported in the first column of Table 2. The results show that the path from pre‐social skills to relationship quality was not significant. Relationship quality, however, was significantly related to post‐social skills, indicating that higher relationship quality was related to higher social skills post mentoring. Gender was significantly related to post‐social skills, which means that girls had higher social skills after the intervention compared to boys. Relationship quality did not mediate the relation between pre‐ and post‐social skills; that is, there was no significant indirect effect of pre‐social skills on post‐social skills via relationship quality in the overall sample, *b *=* *0.009, *SE = *0.01*,* 95% CI [−0.0003, 0.03].

**Table 2:** Unstandardized regression coefficients for the mediated path models between social skills and relationship quality

	Overall mediation	Moderated mediation Gender	Moderated mediation Age
	*b* (*SE*)	*b* (*SE*)	*b* (*SE*)
Paths to relationship quality			
Pre‐social skills	0.16 (0.09)	0.17 (0.10)	0.16 (0.09)
Age	−0.05 (0.03)	−0.05 (0.03)	−0.05 (0.03)*
Gender (1 = girls)	0.12 (0.08)	0.12 (0.09)	0.12 (0.08)
Pre‐social skills × age			−0.12 (0.05)*
Pre‐social skills × gender		−0.21(0.19)	
Paths to post‐social skills			
Relationship quality	0.06 (0.02)**	0.06 (0.03)*	0.06 (0.02)*
Pre‐socials skills	0.61 (0.04)**	0.61 (0.05)**	0.60 (0.05)**
Age	−0.01(0.01)	−0.01 (0.1)	−0.02 (0.01)
Gender (1 = girls)	0.10 (0.04)**	0.10 (0.04)**	0.10 (0.04)**
Pre‐social skills × age			−0.01 (0.03)
Pre‐social skills × gender		0.10 (0.11)	
Relationship quality × age			−0.05 (0.02)**
Relationship quality × gender		−0.05 (0.05)	
	*R* ^2^ = 0.45	*R* ^2^ = 0.47	*R* ^2^ = 0.47

*p < .05. **p < .01.

#### Moderated Mediation with Gender

Although no mediation occurred for the overall sample, we tested if mediation was present under certain conditions, that is, for a specific subsample. As such, we performed a conditional process analysis to assess the moderating role of gender in the direct and indirect paths between social skills and relationship quality, while controlling for age. The results of this conditional mediation analysis are reported in the second column of Table 2. As in the overall model, there was a significant positive association between relationship quality and post‐social skills, but not between pre‐social skills and relationship quality. There were no gender differences in these associations. Additionally, with the other paths being similar to the paths in the overall mediation analysis, gender did not moderate the indirect path from pre‐social skills to post‐social skills via mentor–mentee relationship quality, indicated by the confidence interval for the index of moderated mediation that included zero (*b *=* *−0.02, *SE* = 0.02, 95% CI [−0.08, 0.006]).

#### Moderated Mediation with Age

**Figure 1:**
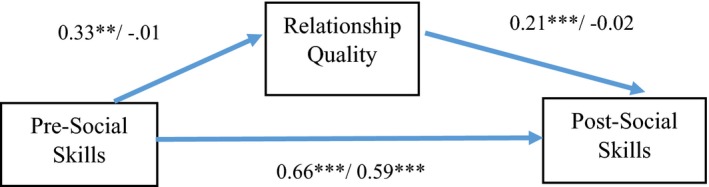
Unstandardized regression coefficients for the mediation paths, controlled for gender. Before the dash for younger mentees (1 *SD* below the mean) and behind the dash for older mentees (1 *SD* above the mean). *p < .05. **p < .01. ***p < .001. [Color figure can be viewed at http://www.wileyonlinelibrary.com/]

Lastly, we ran the previous conditional process analysis with age as the moderator, while controlling for the effects of gender (see third column of Table 2). Pre‐social skills, again, were not associated with relationship quality, but age moderated this path (*b *=* *−0.115, *SE *=* *0.05, *p *=* *.02). Simple effect analyses showed that pre‐social skills were significantly associated with relationship quality for younger mentees (as defined by 1 *SD* below the mean, 55% of the sample), *b *=* *0.33, *t(*367) = 3.05, *p *=* *.002, but not for older mentees (1 *SD* above the mean age) (see Fig. 1). Relationship quality, then, was significantly related to post‐social skills. Moreover, also the path between relationship quality and post‐social skills was moderated by age, *b *=* *−0.049, *SE *=* *0.02, *p *=* *.01. Simple effect analyses showed that the association between relationship quality and post‐social skills was only significant for younger and not older mentees, *b *=* *0.21, *t(*374) = 4.55, *p<*.001 (see Fig. 1). Lastly, the overall mediation model showed that there was a significant indirect effect of pre‐social skills on post‐social skills, via relationship quality for younger mentees, (*b *=* *0.043, *SE *=* *0.019, 95% CI [0.013, 0.090]) but not for older mentees (*b *=* *0.0001, *SE *=* *0.005, 95% CI [−0.009, 0.011]). Thus, for younger aged mentees, the association between pre‐ and post‐social skills is mediated by relationship quality.

## Discussion

The current study aimed to explore how mentees’ social skills before mentoring, mentor–mentee relationship quality, and social skills after mentoring are related in a school‐based mentoring program. Results suggest that only young mentees’ pre‐social skills are associated to mentor–mentee relationship quality. There was, however, a significant positive association between mentor–mentee relationship quality and post‐social skills for the overall sample. Relationship quality did not mediate the association between pre‐social skills and post‐social skills in the overall sample, but for younger mentees, relationship quality did partially explain the association between pre‐social skills and post‐social skills.

### Pre‐social Skills and Relationship Quality

We found support for the hypothesis that mentees with higher pre‐social skills also report higher relationship quality with their mentor, but this was only true for young mentees (age 11–13). For younger mentees, this finding is in accordance with theories and research on the relation between social skills and the quality of interpersonal relationships (Segrin & Taylor, 2007; Segrin et al., 2016). The findings of this study show that for young mentees in school‐based mentoring, and their social skills are related to relationship quality. For older mentees (age 13–19), we did not find this association. Although there were no significant mean differences in perceived relationship quality between younger and older aged mentees, different predictors of relationship quality for both groups may be at play. Youths’ developmental life stage is likely to play a part in determining different needs in mentoring (Allen & Eby, 2007). Qualitative research on the perceptions of mentoring of early to mid and late adolescents revealed differences in how mentees in different developmental stages draw support from their mentor. Younger mentees, for example, were looking up to their mentor, whereas older mentees emphasized mutuality and a need to be on equal footing with their mentor (Liang, Spencer, Brogan, & Corral, 2008). It may be that older mentees’ social skills are less predictive of relationship quality, because of their developmental stage and associated needs concerning mentoring.

### Relationship Quality and Post‐social Skills

Subsequently, we tested the associations between relationship quality and post‐social skills. We found significant associations between relationship quality and post‐social skills, and this association was even stronger for younger than older mentees. The finding that higher relationship quality is associated to higher post‐social skills is consistent with our hypothesis. Previous research on mentoring identified relationship quality as a key factor in mentoring in general (Eby et al., 2013), and in school‐based mentoring in particular (Bayer et al., 2013). That mentor–mentee relationship quality is related to social skills outcomes in school‐based mentoring specifically, confirms the status of relationship quality as a key factor, and is a valuable addition to the extant mentoring literature. One of the measured aspects of relationship quality, role modeling, might explain the relevance of relationship quality for social skills. Research shows that when adolescent peers display prosocial behaviors, adolescents are likely to respond in a prosocial manner. This, in turn, might lead one to engage in cycles of prosocial exchanges (Bukowski & Sippola, 1996). Due to the relatively small age difference between mentor and mentee, the mentor may serve as a role model of skills which explains the association between relationship quality and social skills outcomes.

### Mediating Role of Relationship Quality

Lastly, to explain the mechanism underlying mentoring, we tested the mediating role of mentor–mentee relationship quality in social skills before and after a mentoring program. In the overall sample, relationship quality did not explain the association between pre‐social skills and post‐social skills. The same was true for the model where we tested gender differences, meaning there were no significant differences between boys and girls in the mediating role of relationship quality. However, looking at different age groups, the mediating role of relationship quality varied across age. For younger mentees, relationship quality partially explained the association between pre‐ and post‐social skills. Thus, our results imply that one of the key aspects of mentoring, that is, high relationship quality, may be particularly important for younger mentees, as compared to older mentees. One of the main developmental tasks during adolescence is moving away from parents and developing a new social network (Eccles, 1999; Segrin et al., 2016). For the youngest mentees in our study (i.e., early adolescents aged 11–13), the mentoring intervention might be one of the first times they are developing a one‐to‐one relationship with an older, non‐familial member. With that in mind, having a satisfying and trusting relationship may be, not surprisingly, of greater importance for them as compared to older mentees to accept the mentors’ guidance, role modeling, and direct instructions. As a result, their mentee–mentor relationship quality partially explains the way they develop their social skills along the mentoring program.

### Age and Gender Differences

The results presented above suggest that for younger mentees, relationship quality is important in mentoring‐related changes in their social skills. The proposed gender differences in the associations between social skills and relationship quality were not present. Social skills were not more important for girls’ relationship quality compared to boys (cf. Pollack, 1999), and neither was girls’ relationship quality more important for post‐social skills compared to boys (cf. Karcher, 2008). Despite the finding that there are no differences in associations between the study variables for boys and girls, we did find some mean differences in social skills. Girls reported higher social skills after the mentoring intervention compared to boys. This finding is consistent with our hypothesis and other research on gender development, stating that due to faster neurological maturation and gender role identification, adolescent girls may have higher social skills than adolescent boys (Silberman & Snarey, 1993). Interestingly, inconsistent with studies that showed neurological maturation during adolescence to be linked to increased social skills (Crone & Dahl, 2012), in the current study younger and not older mentees reported higher social skills. Research showed that for boys, although their interpersonal competence is increasing with age, they tend to engage in less social behavior due to their gender role ideology becoming more stereotypically (Flannery & Smith, 2017). This might explain the lower scores on social skills for older boys, which refers to either their actual behavior or the way they self‐reported their behavior. Additionally, we expected girls to report lower relationship quality than boys, given the short‐term character of the mentoring program. However, girls did not report lower relationship quality in the current short‐term school‐based mentoring program, in contrast to previous research on gender differences in relationship quality in community‐based mentoring (Rhodes et al., 2008). Despite the short‐term character of the mentoring program in our study, the structured one‐to‐one, weekly meetings between mentor and mentee, may lead to higher relationship quality for girls compared to community‐based mentoring. At the start of the mentoring intervention, mentees were asked to formulate goals, and this could have stimulated girls to formulate more instrumental goals. This may then result in more realistic expectations of girls’ mentoring relation and lead to higher relationship quality. Future research should test this assumption.

### Strengths and Limitations

Several limitations of this study should be acknowledged. Firstly, creaming may have occurred in the selection of classrooms to enter the mentor program (Lipsky, 2010). Classrooms were not randomly assigned to the mentoring condition, but schools decided which classrooms were entered in the mentor program. It could be the case that schools assigned classrooms in with students who were more open to mentoring to the mentor condition, which, therefore, were more likely to succeed. Secondly, as the majority of the mentoring took place in the intended one‐on‐one situation, we ascribe found effects to this particular type of mentoring. However, the effect might be somewhat distorted by the fact that some mentees occasionally received group mentoring (one mentor, two mentees). For example, perceived relationship quality might be depending on the fellow mentees’ social skills instead of on the mentees’ own socials skills. Thirdly, mentees who were part of our final sample had higher post‐social skills than mentees who did not complete all the surveys. However, mentees did not differ in pre‐social skills and mentor–mentee relationship quality, but it could still indicate that our subsample was somewhat more “successful” in terms of desired mentoring‐related outcomes (i.e., social skills). Lastly, we used self‐reported measures of social skills. This may give an inaccurate impression of youths’ actual social skills. On the one hand, mentees could have overestimated their social skills due to a lack of self‐insight or social desirability. On the other hand, for boys, social skills might increasingly become less desired with increasing age, and therefore, self‐reports might give an underestimation (Flannery & Smith, 2017).

Despite these limitations, the current study is, to the best of our knowledge, the first to examine a school‐based mentoring program in the Netherlands, taking into account age and gender differences. We focused on social skills as a facilitating factor of relationship quality, contributing to the base of knowledge of mentoring. Our results imply that school‐based mentoring is most beneficial for younger students and that their mentor–mentee relationship quality is important in developing social skills.

## Implications for Future Research

More knowledge is necessary on which subgroups of youths are more likely to benefit from school‐based mentoring. Since this study showed that young mentees’ social skills were related to mentor–mentee relationship quality, further research is needed on what factors are related to relationship quality in mentoring for older mentees. The present study only used youth characteristics in explaining relationship quality, but mentor characteristics have been found to partially account for relationship quality as well. To illustrate, mentors’ self‐disclosure, self‐efficacy, goal‐setting, feedback, previous involvement with youths, and mentors’ experiences with the program are related with relationship quality (Dutton, 2018; Lyons, McQuillin, & Henderson, 2019; Raposa, Rhodes, & Herrera, 2016; Weiler, Boat, & Haddock, 2019). The structured content of the evaluated mentoring program guided mentors and mentees in their activities. In many mentoring practices, however, programs only provide general guidelines of how to develop a constructive relationship. This might influence the way mentors and mentees establish a fruitful relationship and, as a result, their relationship quality. Therefore, further research is needed to see whether the finding that young mentees’ social skills are related to relationship quality is also true for other school‐based mentoring programs and for community‐based mentoring.

Relationship quality in general appeared to be related to social skills. Therefore, before future research is able to identify individual characteristics that influence relationship quality, school‐based mentoring programs should focus on providing the right conditions to increase this relationship quality, such as facilitating weekly meetings and opportunities to interact outside a large group setting (Bayer et al., 2013).

## Conclusions

In sum, this study showed that higher relationship quality is related to higher social skills after mentoring and that only for younger students, social skills pre‐intervention are related to relationship quality. Finally, mentor–mentee relationship quality explains the relation between young mentees’ pre‐ and post‐social skills. These results suggest that, for social skills to improve, school‐based mentoring programs should pay close attention to mentees’ abilities to develop positive (mentoring) relationships.

## Conflict of Interest

The authors have no conflicts of interest to disclose.
